# Multiple independent chromosomal fusions accompanied the radiation of the Antarctic teleost genus *Trematomus* (Notothenioidei:Nototheniidae)

**DOI:** 10.1186/s12862-020-1600-3

**Published:** 2020-03-20

**Authors:** Juliette Auvinet, Paula Graça, Agnès Dettai, Angel Amores, John H. Postlethwait, H. William Detrich, Catherine Ozouf-Costaz, Olivier Coriton, Dominique Higuet

**Affiliations:** 1 Sorbonne Université, CNRS, Université des Antilles, Evolution Paris Seine - Institut de Biologie Paris Seine (EPS - IBPS), 75005 Paris, France; 2 Institut de Systématique, Evolution, Biodiversité (ISYEB) – Muséum National d’Histoire Naturelle, CNRS, Sorbonne Université, EPHE, Université des Antilles, 43, rue Cuvier, 75231 Paris Cedex 05, France; 3 Department of Marine and Environmental Sciences, Marine Science Center, Northeastern University, Nahant, MA 01908 USA; 4 Institute of Neuroscience, Department of Biology, University of Oregon, Eugene, OR 97403 USA; 5 Institut National de Recherche pour l’agriculture, l’alimentation et l’environnement, INRAE, UMR1349 IGEPP, Molecular cytogenetics Platform, BP35327, F-35653 Le Rheu Cedex, France

**Keywords:** BAC-FISH, Chromosomal painting, Chromosomal rearrangements, Chromosomal structural units, Chromosomal synteny, Nototheniidae, Speciation

## Abstract

**Background:**

Chromosomal rearrangements are thought to be an important driving force underlying lineage diversification, but their link to speciation continues to be debated. Antarctic teleost fish of the family Nototheniidae (Notothenioidei) diversified in a changing environmental context, which led to ecological, morphological, and genetic differentiation among populations. In addition, extensive chromosomal repatterning accompanied species divergence in several clades. The most striking karyotypic changes involved the recent species radiation (about 10 *My*) of the genus *Trematomus*, with chromosomal pair numbers ranging between 29 and 12. These dramatic reductions in chromosome number resulted mostly from large-scale chromosome fusions. Multiple centric and/or tandem fusions have been hypothesized in at least seven of the twelve recognized *Trematomus* species. To reconstruct their evolutionary history, we employed comparative cytogenomics (BAC-FISH and chromosome painting) to reveal patterns of interspecific chromosomal orthologies across several notothenioid clades.

**Results:**

We defined orthologous chromosomal segments of reference, termed Structural Units (SUs). SUs were identified in a total of 18 notothenioid species. We demonstrated for the first time that SUs were strongly conserved across every specimen examined, with chromosomal syntenies highlighting a paucity of intrachromosomal macro-rearrangements. Multiple independent fusions of these SUs were inferred in the *Trematomus* species, in contrast to the shared SU fusions in species of the sister lineage *Notothenia*.

**Conclusions:**

The SU segments were defined units of chromosomal rearrangement in the entire family Nototheiidae, which diverged from the other notothenioid families 20 *My* ago. Some of the identified chromosomal syntenies within the SUs were even conserved in their closest relatives, the family Eleginopsidae. Comparing the timing of acquisition of the fusions in the closely related genera *Notothenia* and *Trematomus* of the nototheniid species family, we conclude that they exhibit distinct chromosomal evolutionary histories, which may be relevant to different speciation scenarios.

## Background

During the last 20 million years (*My*), several glacial-interglacial cycles caused strong pulsatory environmental changes in the Southern Ocean. Glacial maxima on the Antarctic continental shelf and peri-insular plateau led to habitat disturbance, including iceberg scouring and habitat fragmentation [1–5]. In this cooling and fluctuating environment, the monophyletic “Antarctic clade” of the nototheniid fish (Notothenioidei:Nototheniidae^1^) adapted via biochemical and physiological innovations (e.g., evolution of the protective antifreeze glycoproteins or AFGPs [9–13]). Their diversification involved several rounds of species radiation events [6, 14–18], which led to ecological, species-specific, and genetic differentiation [16, 19–21]. In some clades, chromosomal changes accompanied lineage diversification [22–25]. Because fixation of chromosomal change can lead to reproductive isolation [26, 27], characterization of these chromosomal repatterning events is an important step toward reconstruction of the evolutionary history of the Antarctic teleosts [13, 20, 28].

Reconstruction of the ancestral vertebrate karyotype (*n* = 10, 12 or 17) [29–31] led to the *n* = 24 or 25 inferred teleost “proto-karyotype,” resulting from a complete genomic duplication (TGD for the Teleost Genome Duplication) [29–38] and dated between 316 and 226 *My* [29, 39–42]. Great chromosomal stability has generally been found since the TGD in various teleost fish taxa [25, 43, 44], although some species deviate slightly from *n* = 24–25 (including cod, *Gadus morhua*, *n* = 23 [45]; fugu, *Takifugu rubripes*, *n* = 22 [46]; sticklebacks, *Gasterosteus aculeatus*, *n* = 21 and *Apeltes quadracus, n* = 23 [47, 48]; and tetraodon, *Tetraodon nigroviridis*, *n* = 21 [49]).

The “proto-karyotype” of *n* = 24 acrocentrics has been hypothesized to be the plesiomorphic state for the notothenioid sub-order [50, 51], because *n* = 24 is found in the families Bovichtidae [50, 52], Pseudaphritidae, and Eleginopsidae [52, 53], the latter being the closest taxon to the Nototheniidae. Many sub-families of the mostly Antarctic endemic family Nototheniidae have quite conserved karyotypes (Channichthyinae: *n* = 24 [22, 54], Artedidraconinae: *n* = 23 [24, 55, 56]), whereas other sub-families display high variability in chromosome numbers. The most striking chromosomal diversity has been found within the genus *Trematomus* of the sub-family Trematominae [23, 24, 57], in which haploid chromosome numbers range from 29 to 12 [51, 57]. Besides, species in the genus *Notothenia* of the Nototheniinae, have also experienced massive chromosomal reductions in karyotypes (*n* = 11, 12 and 13) [22, 24, 25]. Karyotypic variability in the Nototheniidae has also been documented within single species in these genera [22–24, 57, 58]. Pericentric inversions, fusions and fissions of chromosomes and chromosomal segments, mediated by mobilization of retrotransposons [59, 60], have been hypothesized to be the most common causes of chromosomal diversification in *Trematomus* and *Notothenia* [22, 23, 51, 61]. Thus, genomic rearrangements, which are known to result in post-zygotic gametic incompatibilities, may have accompanied speciation events within the Nototheniidae [27, 62–65].

To identify chromosome rearrangement events occurring during nototheniid diversification, interspecific chromosomal homologies (ICHs) must be characterized. To overcome the difficulties in correctly comparing sizes and morphologies among nototheniid chromosomes, we determined a chromosomal unit of reference called a Structural Unit (SU) based on the karyotype of the platyfish, *Xiphophorus maculatus*, and transposed via synteny to the karyotype of the three-spine stickleback, *G. aculeatus*, and the Antarctic bulhead notothen, *Notothenia coriiceps* by [25]). SUs of *Notothenia* and *Trematomus* species were then identified by Fluorescence In Situ Hybridization (FISH) of BAC-clones containing genomic DNA of *N. coriiceps* [66–68] and by chromosomal painting. Results show that chromosomal fusions in *Trematomus* occurred independently in different species lineages, unlike the mostly shared fusions of *Notothenia* species, highlighting contrasting evolutionary histories between genera within the Nototheniidae.

## Results

### Broad conservation of structural units (SUs) across notothenioid clades

#### Identifying SU markers: hybridization of N.coriiceps BACs to *N. coriiceps* chromosomes

**Fig. 1:**
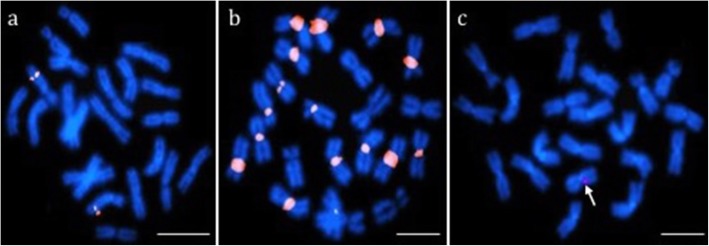
BAC hybridization signals on chromosomes of *N. coriiceps*. **a** Specific signal: Strong, discrete signal on a single chromosome pair (example with BAC M11). **b** Repetitive signal: Repetitive, typically centromeric/pericentromeric signal on multiple chromosome pairs (example with BAC B9). **c** Weak signal: Weak signal, rarely detected on chromosomes (example with BAC •J2). Only BAC probes producing specific signals were used for subsequent analyses. Hybridization patterns were independent of the fluorochrome used for detection. Scale bars: 10 μm

**Fig. 2:**
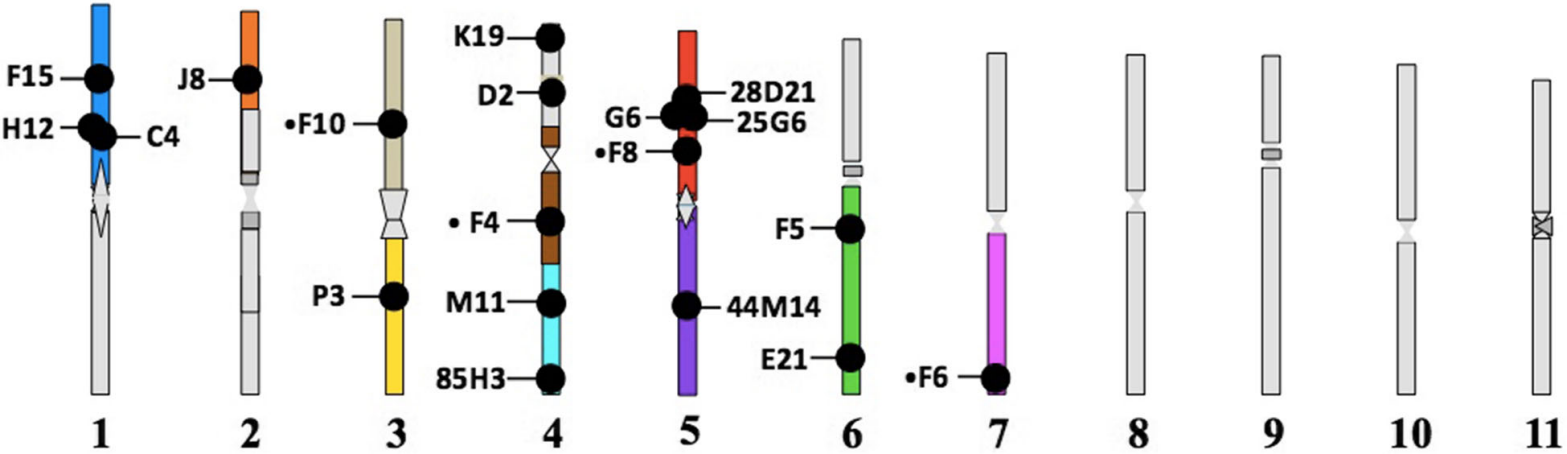
Mapping of “specific” BACs to the haploid chromosome set of *N. coriiceps*. Chromosomes were classified by their relative size. Some were recognizable by their DAPI-counterstaining patterns. Each arm corresponds to a Structural Unit (SU), with the exception of chromosome pair number 2 and 4, which contain three SUs [25]. Each SU identified by BAC labeling is represented by a unique color (Additional file 1)

Our first goal was to develop BAC-FISH probes for the teleost SUs defined in Amores et al. [25]) based on comparative genetic mapping of the genomes and karyotypes of *X. maculatus*, *G. aculeatus*, and *N. coriiceps*. We selected 40 *N. coriiceps* BACs at random and screened 37 by hybridization to chromosome preparations of *N. coriiceps*. The remaining three were hybridized only on *Trematomus* species chromosomes. Results revealed three main types of fluorescent signals (Additional file 1). Twenty-two BACs (55% of total), including 19 BACs in *N. coriiceps*, gave clearly discrete double spots at a single location on one chromosomal pair (“specific” signals; Fig. 1a), corresponding to specific hybridization to unique chromosomal sequences. The second pattern involved broader bands detected on multiple chromosome pairs and usually in centromeric/pericentromeric regions (“repetitive”, Fig. 1b) and was found for seven BACs (17.5%). These multiple hybridization signals were probably due to high proportions of repetitive sequences (e.g., transposable elements, satellites, etc.) that were not blocked despite prehybridization with competitor and carrier DNAs. The third signal category (“weak” signals; 11 BACs = 27.5%) presented as weak spots (single or multiple) in nuclei that were rarely visible on metaphasic chromosomes (Fig. 1c). These weak signals could be due to genomic content of the BAC itself, or to defective probe labeling. Only the 22 BACs that produced the specific signals were selected for subsequent chromosomal analyses (Fig. 2).

#### Conservation of structural units across nototheniid species

To test whether genetic synteny is conserved between the SUs defined for *N. coriiceps* and the chromosome sets of other notothenioid species, we hybridized BACs in various combinations to chromosome preparations from species representing the Trematominae, Dissostichinae, Channichthyinae, Cygnodraconinae and Gymnodraconinae. First, we used two or more SU-specific BACs to evaluate marker positions and orientations relative to centromeres and telomeres and to evaluate inter-marker distances. Fig. 2 shows that five distinct SUs in *N. coriiceps* are defined by multiple BACs: three by two BACs (K19/D2, M11/85H3, F5/E21, respectively), one by three BACs (F15/H12/C4), and one by four BACs (28D21/G6/25G6/•F8).

**Fig. 3:**
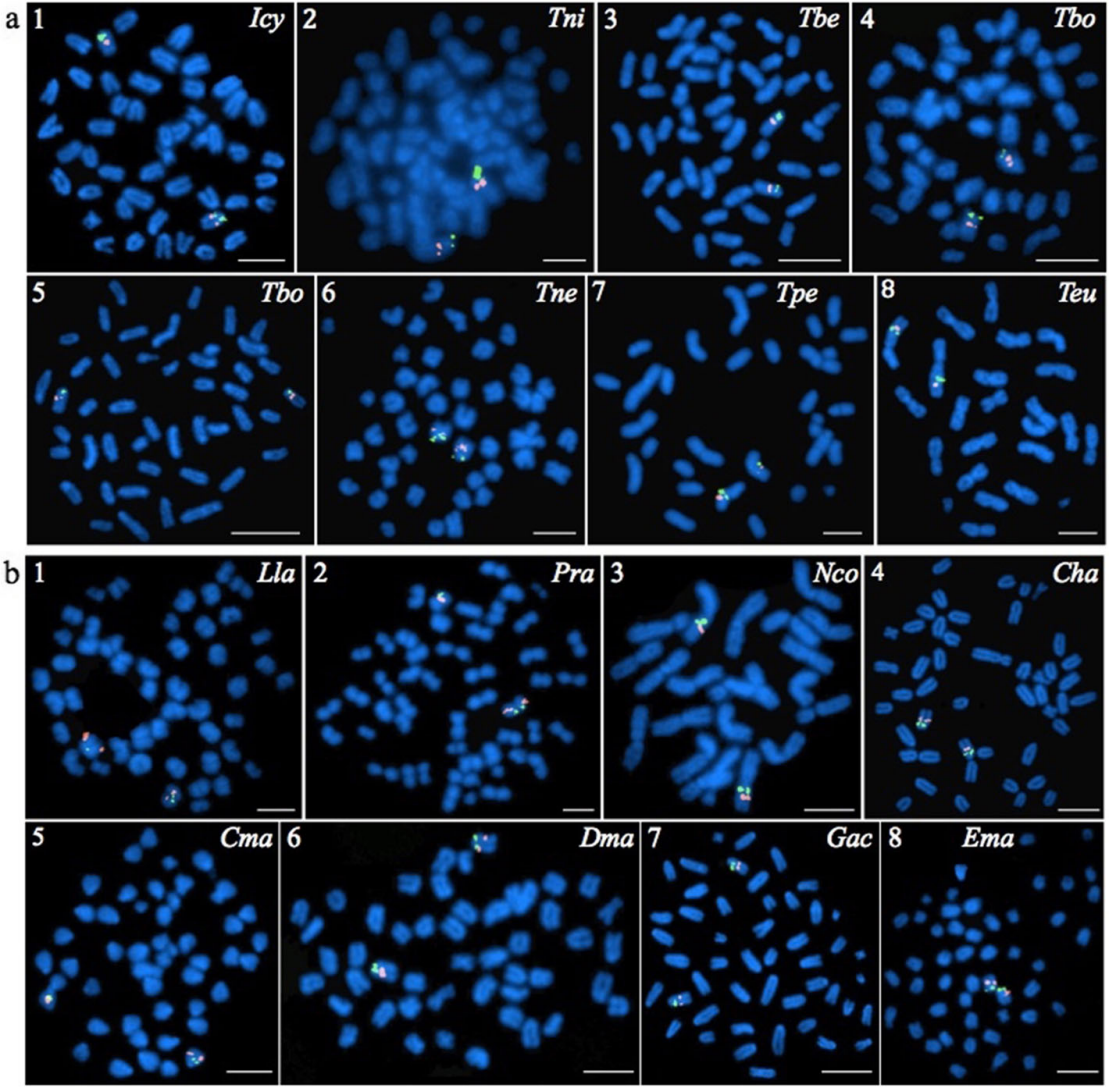
Hybridization of BACs C4 and F15 defines a conserved SU in the Trematominae and other notothenioid clades. a) *Trematomus* species: 1, *I. cyanobrancha* (*Icy*); 2, *T. nicolai* (*Tni*); 3, *T. bernacchii* (*Tbe*); 4, *T. borchgrevinki* (*Tbo*); 5, *T. hansoni* (*Tha*); 6, *T. newnesi* (*Tne*); 7, *T. pennellii* (*Tpe*); and 8, *T. eulepidotus* (*Teu*). b) Eight notothenioid species from other clades: 1, *L. larseni* (*Lla*); 2, *P. ramsayi* (*Pra*); 3, *N. coriiceps* (*Nco*); 4, *C. hamatus* (*Cha*); 5, *C. mawsoni* (*Cma*); 6, *D. mawsoni* (*Dma*); 7, *G. acuticeps* (*Gac*); and 8, *E. maclovinus* (*Ema*). Hybridization of BAC C4 probe was detected using fluorescein (green signals), whereas BAC F15 was imaged using rhodamine (red signals). Chromosomal DNA was counterstained with DAPI (blue). Scale bars: 10 μm

**Fig. 4:**
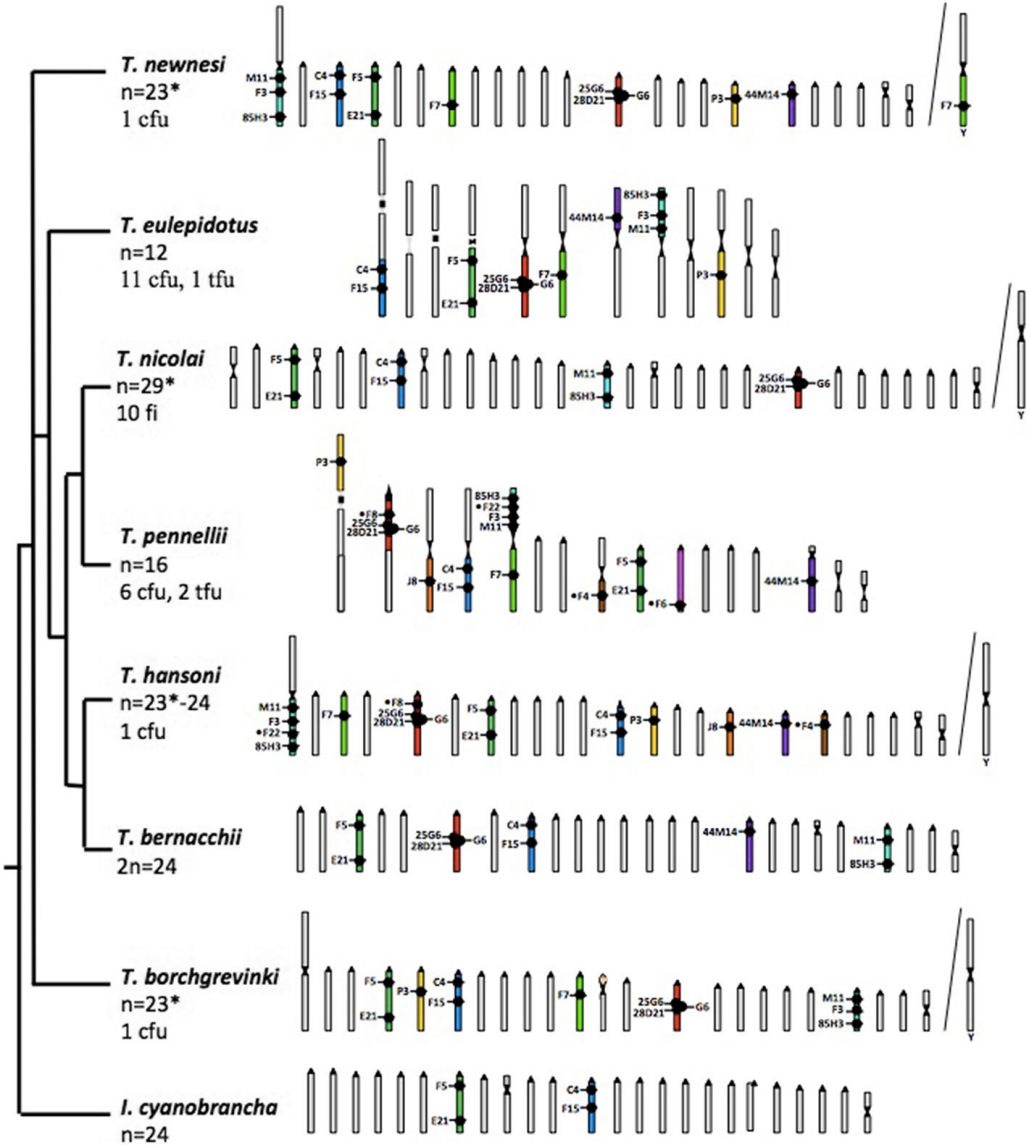
Mapping of BAC-defined SUs onto the idiograms of haploid female chromosome sets of eight *Trematomus* species. Karyotypes with identified SUs and fusions are plotted against the *Trematomus* phylogeny [8]. Orthologous SUs are shown in the same color. Four species (*T. nicolaï*, *T. hansoni*, *T. borchgrevinki* and *T. newnesi*) have sex-differentiated chromosomes (X1X1X2X2 (female) / X1YX2 (male)). The haploid female chromosome number of each is presented, while asterisks indicate when male (chromosomal formulae: 2n-1), and the male chromosome (Y) is shown on the right side of the corresponding karyotype. Total numbers of fusions (cfu, centric fusion; tfu, tandem fusion) or fissions (fi) are indicated (based on female karyotypes) for the six species that deviate from *n* = 24. Specific BAC designations are given next to their locations on chromosomes. Note that BACs F3, F7 and •F22 were not mapped to *N. coriiceps* SUs in Fig. 2

To determine the extent to which SUs are conserved in nototheniids, we hybridized the BAC pair C4 and F15 (chosen randomly among the BAC pairs that delineated a single SU in *N. coriiceps*; Fig. 2) to eight species of *Trematomus* (Fig. 3a, Additional file 1) and eight other notothenioid species (Fig. 3b, Additional file 1). Results showed that the relative positions of the two markers mapped to single chromosome arms of all species and that the distances between them were generally well conserved. Thus, C4 and F15 define an orthologous SU across the 16 species, which include two Sub-Antarctic species (*E. maclovinus*, *P. ramsayi*). Furthermore, BACs 28D21, C4, and M11 consistently labeled near pericentromeric regions, whereas BACs E21 and 85H3 were found near telomeric regions of their respective SUs (Fig. 2). However, labeling by BAC 44 M14 was variable, being found in the middle of a SU of *N. coriiceps* (Fig. 2) and *T. pennellii* (Fig. 4), but in pericentromeric regions of a SU in *T. bernacchii*, *T. hansoni*, *T. newnesi*, and *T. eulepidotus* (Fig. 4). Such changes in location with respect to the centromere could be explained by either pericentric or paracentric inversions.

The relative distances between BAC hybridization locations were also conserved across species, again exemplified by centromeric BAC C4 and interstitial BAC F15 (Figs. 3 and 4). Similarly, pericentromeric BAC F5 and sub-telomeric BAC E21 were located in the same SU and separated by equivalent distances in the eight *Trematomus* species (Fig. 4).

**Fig. 5:**
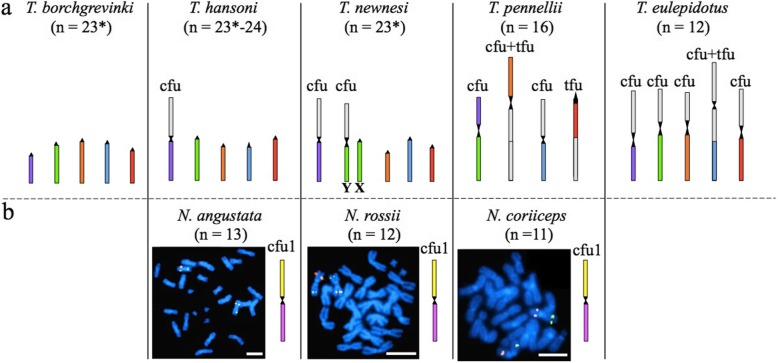
Comparison of chromosomal fusions in the genera *Trematomus* and *Notothenia*. A subset of BAC-defined SUs was used to characterize chromosomal fusion (cfu: centric fusions, tfu: tandem fusion). Each distinct SU is represented by a unique color. Orthologous SUs are labeled by the same color. **a***Trematomus* species. Independent chromosomal fusions were observed in the five species shown. The asterisks indicate when male (chromosomal formulae: 2n-1). The green SU was found in the heteromorphic Y sex chromosome in males in *T. newnesi*, which corresponds to the X1 or X2 sex chromosome in females. *T. borchgrevinki* and *T. hansoni* were represented only by females in this study (Additional file 2). **b***Notothenia* species. BAC pair 28D21 (green signal) and 44 M14 (red signal), which label different SUs (Additional file 1), revealed that orthologous SUs formed the same metacentric pair in the three *Notothenia* species (cfu1 = shared fusion). Abbreviations: cfu; centric fusion; tfu, tandem fusion. Scale bars: 10 μm

Recognizing the high degree of interspecific conservation of chromosomal synteny among the notothenioids [25, 69], we also used six BACs that hybridized as singletons to *N. coriiceps* SUs (Fig. 2) to infer SUs in other species. In these cases, we explicitly assume that a single BAC signal indicates the conservation of an entire SU, at least at the chromosomal scale (macro-rearrangements). Conserved synteny could even be extrapolated to the whole SU for cases we studied. Six additional SUs were labeled by a unique BAC in species of the genus *Trematomus* (Fig. 4), and two additional SUs in species of the genus *Notothenia* (Fig. 5).

### Chromosome fusions in the Nototheniidae: independent events in *Trematomus*, shared events in *Notothenia*

The Nototheniidae are notable for their diverse karyotypes, with both fusions and fissions altering the canonical teleost chromosome set of *n* = 24 [50, 51]. Regarding fusions, for which we have the most data in the genera *Trematomus* and the *Notothenia* (Figs. 2, 3, 4, 5), one may ask: did they occur independently among the species of these clades? Or are they shared by virtue of descent from a common ancestor? Surprisingly, the answer appears to be taxon-specific. Results showed that an overwhelming majority of the centric and/or tandem fusions detected in this study were not shared among *Trematomus* species (Fig. 4). For example, the largest sub-metacentric pairs of *T. pennellii* and *T. eulepidotus*, each of which probably arose by a combination of centric and tandem fusion events [27], contained non-orthologous SUs (orange and blue, respectively; Figs. 4, 5a). The red SU (defined by BACs 28D21, 25G6, G6 and •F8) was fused tandemly in *T. pennellii* but formed a centric fusion in *T. eulepidotus* (Figs. 4, 5a). The green and purple SUs (marked by BACs F7 and 44 M14, respectively) partnered in a centric fusion in *T. pennellii*, whereas they were involved in centric fusions with different SUs in *T. hansoni*, *T. newnesi* and *T. eulepidotus* (Fig. 5a). We conclude that many, if not all, of the chromosomal fusion events in the *Trematomus* radiation occurred independently.

Throughout the trematomine species examined, the SUs defined by BACs 28D21 and 44 M14 were found in two different chromosomes. In striking contrast, these SUs were fused centrically to form the same orthologous metacentric chromosome pair in the three species of the genus *Notothenia* (Fig. 5b), as shown by the relative positions of the BACs on their SUs. Our observation supports prior results obtained by genetic mapping [25], that demonstrate that chromosomal fusions in this genus are generally shared across the three species.

### Identification of nototheniid interspecific chromosomal homologies (ICHs) by chromosomal painting

**Fig. 6:**
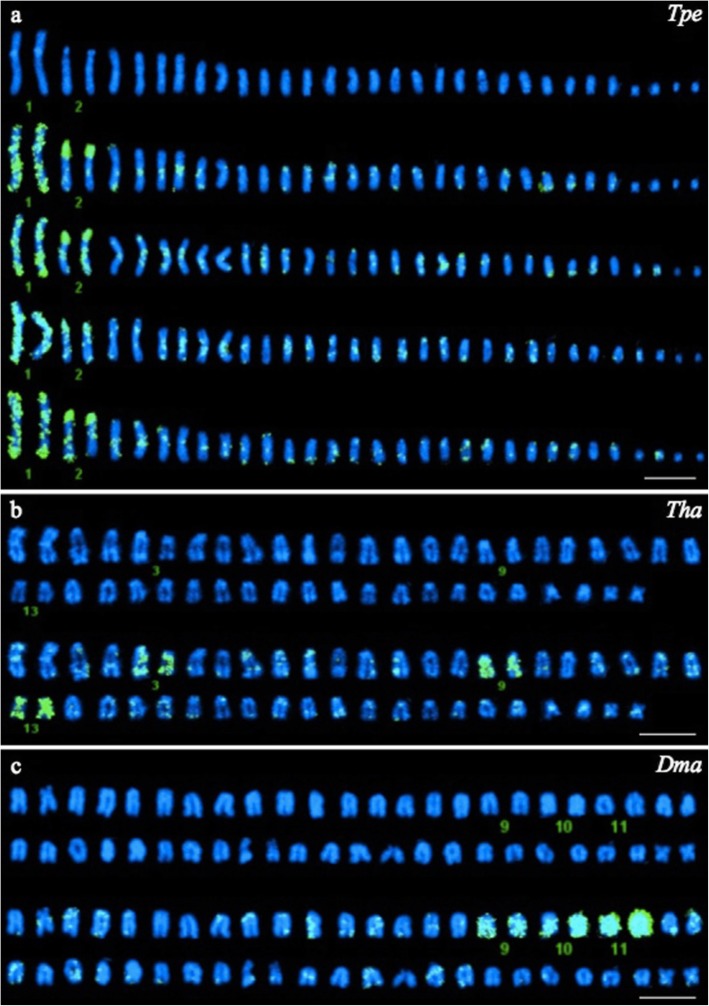
Chromosome 1 of *T. pennellii* is a fusion product of three ancestral chromosomes. **a** Positive control: Hybridization of the *T. pennellii* painting probe to *T. pennellii* chromosomes (*n* = 16). **b** and **c** Hybridization of the *T. pennellii* probe to chromosomes from *T. hansoni* (Tha, *n* = 23) and from *D. mawsoni* (*n* = 24), respectively. Each panel shows a DAPI-stained karyotype at the top, and one (b, c) or more (a) karyotypes after hybridization of the painting probe. Bound painting probe was imaged by FISH, and detected using fluorescein (green signals). Numbered chromosome pairs are referenced in the text. Scale bars: 10 μm

We hypothesize that the uniquely identifiable largest sub-metacentric pair of *T. pennellii* arose by the fusion of several SUs. To test that hypothesis, we used a painting probe prepared from *T. pennellii* chromosome 1 to search for interspecific chromosomal homologies in other notothenioid species. Hybridization of the *T. pennellii* probe to chromosome spreads from *T. pennellii* gave the expected robust, full-length hybridization to chromosome pair 1 (Fig. 6a). In addition, chromosome pair 2, the second largest and acrocentric chromosome, showed intense and uniform hybridization near the centromere and some punctate staining elsewhere. Other chromosome pairs were characterized by weak staining of small, often telomeric regions. These nonspecific signals probably correspond to repetitive elements present in the probe, such as *DIRS1* retrotransposons [59].

Interspecific hybridization of the *T. pennellii* chromosome 1 painting probe to chromosomes of *T. hansoni* and *D. mawsoni* labeled three small acrocentric pairs in both species (Figs. 6b, c): 3, 9 and 13 for the former, and 9, 10, and 11 for the latter. Therefore, chromosome pair 1 of *T. pennellii* results from a centric and a tandem fusion involving three SUs (Figs. 4 and 5) inherited from the most recent common ancestor of the Nototheniidae. Nonspecific low level signals like those observed in *T. pennellii* were also dispersed on other chromosomes.

## Discussion

### Sequence conservation in notothenioid fish

**Fig. 7:**
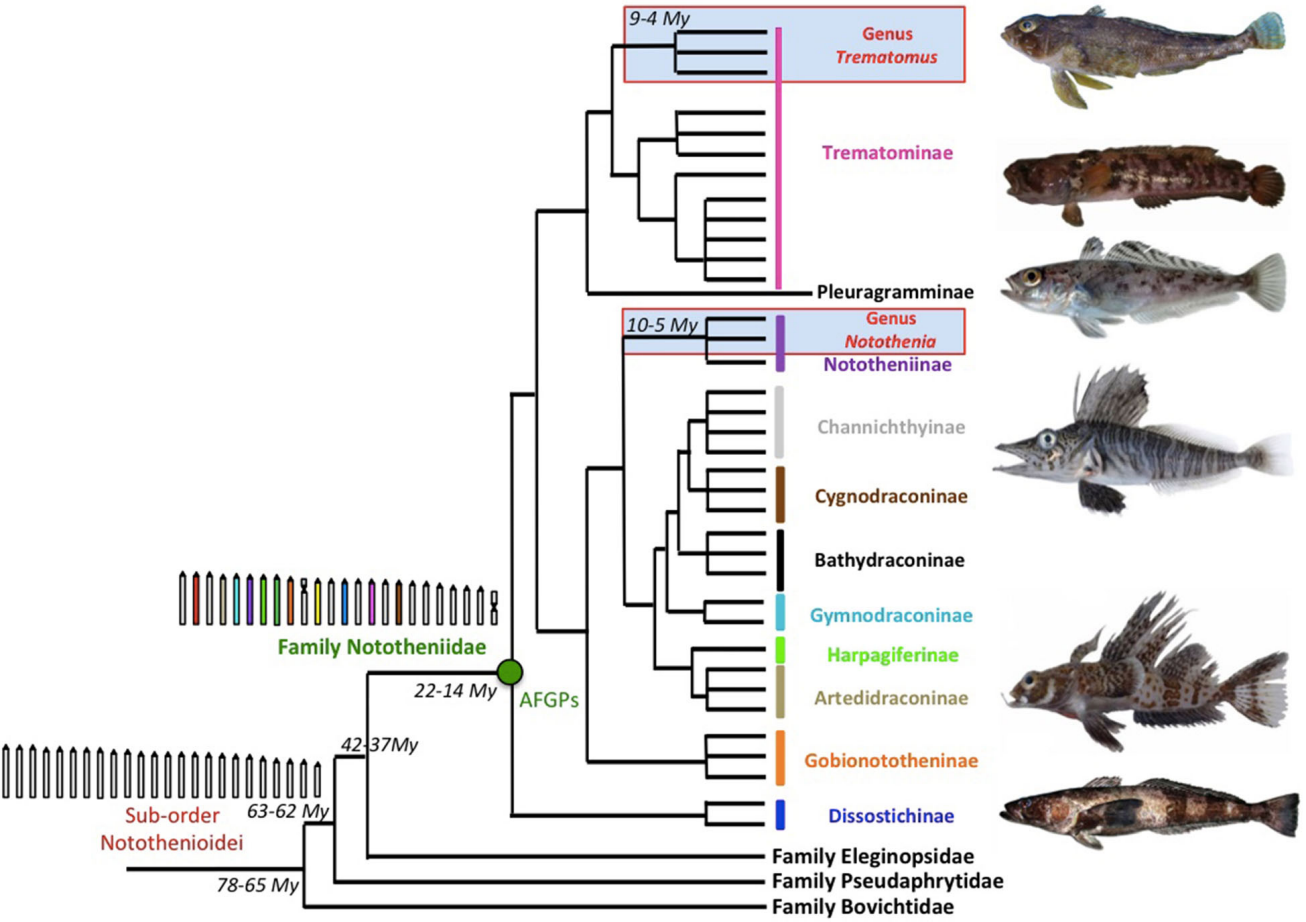
Phylogenetic relationships within the suborder Notothenioidei with inferred ancestral karyotypes**.** Phylogenetic relationships of the notothenioid fish and the nototheniid sub-families and times of divergence according to recent literature [14–18]. Conservation of the 24 defined SUs across nototheniid species and genera allowed the reconstruction of their inferred ancestral karyotype. The reconstitution of this plesiomorphic state was based on the BAC positions on chromosomes of species in the genera *Trematomus* and *Notothenia* (underlined in blue) and the relative size of the SUs identified. In the reconstructed ancestor, all chromosome pairs are acrocentric, except for two SUs that are sub-metacentric and metacentric due to pericentric inversions acquired in the last common ancestor of the families Pseudaphritidae, Eleginopsidae, and Nototheniidae [50, 52, 53, 61]. The inferred ancestral karyotype of the sub-order Notothenioidei is composed of 24 SUs defined as acrocentric chromosomes. We acknowledge S. Iglesias and G. Duhamel (© CEAMARC and POKER surveys, MNHN), and GeShaFish (*D. mawsoni,* Attribution-Share Alike 3.0 Unported license) for the nototheniid photos, from top to bottom: *T. pennellii*, *I. cyanobrancha*, *T. eulepidotus*, *P. macropterus*, *A. orianae*, *D. mawsoni*

Within the suborder Notothenioidei, the family Nototheniidae is both the most speciose [6, 7, 19, 28] and the most prone to chromosomal rearrangements [24, 57, 61]. The specific hybridization of 22 of 40 randomly selected BACs from *N. coriiceps* to 18 representatives within the Nototheniidae suggests, as anticipated, strong sequence conservation within the family. This finding is consistent with the recent divergence time of the family, dated between 22.4 and 13.35 *My* [14, 10] (Fig. 7). Strong conservation across nototheniid genera has already been mentioned for the comparison of *N. coriiceps* and *C. aceratus* [66, 67], and in molecular phylogenetic studies conducted in the whole family [14–17]. We also demonstrated that this conservation can be extended to the sister family of Nototheniidae, the Eleginopsidae, which diverged between 42 and 37 *My* [14, 17] (Fig. 7). We did not detect hybridization of two *N.coriiceps* BACs, C4 and F15, to *Bovichtus diacanthus* from the more distantly related family Bovichtidae (divergence time between 78 and 65 *My* [14, 17] (Fig. 7)).

### Chromosome rearrangement units in Nototheniidae

Strong interspecific conservation of chromosomal segments was identified through BAC combinations that co-localized in the same SU (Figs. 3 and 4). Based on these observations of BAC positions as well as on the chromosomal painting results, the SUs defined in [25] could indeed be the primary units for most rearrangements that occurred during nototheniid species diversification. Those chromosomal units are shared and conserved across nototheniid genera. The SUs would thus have probably been inherited from the last common ancestor of the Nototheniidae (Fig. 7) [23, 24, 51, 57]. This interspecific conservation goes hand in hand with a low number of macro-rearrangements detected within a SU. We did not observe any chromosomal inversion or translocation/transpositions at the SU scale when locating the BACs and identifying ICHs, although the pericentromeric vs interstitial positions of BAC 44 M14 revealed the possibility of local and segmental insertions/deletions (Figs. 2 and 4). This hypothesis is also supported by our painting results (one chromosome of *T. pennellii* corresponds to three acrocentrics in *T. hansoni* and *D. mawsoni*) and corroborated by the absence or low number of secondary rearrangements, as suggested for the genus *Notothenia* by Amores et al. [25].

The various ways in which these SUs are rearranged in the different species generated the karyotype diversity currently observed in this group. This study confirmed the previously hypothesized importance of structural fusions accompanying speciation events, including constant genome size despite changes in chromosome number [23, 51, 59, 61, 66]. Based on a comparison of the total number of chromosomes in nototheniid species, some authors hypothesized a tendency to chromosome number reduction by fusions [23, 51, 55, 57, 61]. The use of BACs to identify orthologous chromosomes in the genera *Trematomus* and *Notothenia*, coupled with observations on chromosome morphology and relative sizes, confirm that chromosome fusions (either Robertsonian fusions or tandem fusions) are the major type of macro-rearrangement event that occurred during chromosomal evolution in this family [23, 25, 51, 57, 61].

### Shared or independent fusions: two contrasting evolutionary histories of karyotype diversification

Comparing the locations of selected BACs in the different nototheniid species enabled us to reconstruct the evolutionary scenario of the fusions that accompanied the diversification of the genera *Trematomus* and *Notothenia*. Because the centric or tandem fusions detected in the present study in the different *Trematomus* species did not involve the same SUs, they resulted from events occurring independently in the different lineages. ICH (interspecific chromosomal homologies) characterization, however, is needed to generalize these observations to all fusions detected in the *Trematomus* karyotypes (Fig. 4). Indeed, if we assume that the fusion involving the purple and the green SUs is specific to *T. pennelli*, then, we still need to identify the SU(s) fused to the purple SU in *T. hansoni*, *T. newnesi* and *T. eulepidotus* to determine whether the corresponding chromosomes are homologous in these species (Figs. 4 and 5).

The evolutionary pattern of karyotype evolution in the genus *Notothenia* completely differed from that of the genus *Trematomus*. Cytogenetic labeling identified one centric fusion (cfu1 in Fig. 5b) shared across the three closely related *Notothenia* species: *N. coriiceps*, *N. rossii* and *N. angustata* [14, 16, 17, 70]. This observation corroborates the evolutionary scenario proposed by Amores et al. [25]) of at least eleven chromosomal fusions acquired in the last common ancestor of *Notothenia* (including also *P. magellanica*). Furthermore, the SUs that fused to form the chromosome pair highlighted in Fig. 5 for *Notothenia* species were never associated together in the *Trematomus* species we examined (*T. eulepidotus*, *T. pennellii*, *T. newnesi* and *T. bernacchii*) (Fig. 4). Therefore, this centric fusion (cfu1 in Fig. 5) is probably not shared between the *Trematomus* and *Notothenia* species.

We could not determine whether all of the chromosome fusions in various *Notothenia* species resulted from a common history. Markers for more SUs would be required to investigate whether every metacentric or submetacentric in *N. coriiceps* (for example, •F10 and P3, or D2 and •F4, Fig. 1) fused with the same partner in other *Notothenia* species. If all fusions were shared across the genus, then the fusion events would have happened at the base of the *Notothenia* diversification before species divergence. It is also possible that additional events occurred after the speciations, such as the translocation between a segment containing the NOR genes in the middle of a sub-metacentric pair in *P. magellanica* and a small acrocentric pair in *N. angustata* (both *n* = 13). Alternatively, some of the fusions could have been acquired independently in some lineages (like one or two sub-metacentric pairs of *N. rossii* (*n* = 12) or *N. coriiceps* (*n* = 11) respectively thought to combine three fused SUs [25]).

### “Chromosomal speciation”?

The fixation of chromosomal fusions in populations constitutes a step toward reproductive isolation [26, 27] because during meiosis the fused chromosome from one population would pair with the two ancestral unfused chromosomes from the other population, leading to the production of aneuploid gametes, to aneuploidy offspring, and presumably to non-viable hybrid progeny. Because they promote the accumulation of genetic incompatibilities, chromosomal fusions may have facilitated lineage-specific diversification in rodents, insects, and also in fishes [71–78]. As proposed previously in the literature, chromosome fusions are the major rearrangement events accompanying nototheniid species diversification, although pericentric inversions and chromosome fissions have also occurred [23, 50–53, 57, 58].

Identifying interspecific chromosomal orthologies in the highly rearranged karyotypes of species in the genus *Trematomus* demonstrated that chromosomal fusions were mostly acquired independently across lineages. We characterized numerous fusions, but none so far were shared in this group, when taking into account the phylogenetic relationships across the different species (Fig. 4), although previous studies hypothesized the sharing of other type of rearrangements [24, 26, 50, 53]. Indeed, the two shared pericentric inversions acquired before the last common ancestor of the Pseudaphritidae, Eleginopsidae, and Nototheniidae families may have produced the chromosomal pair bearing the 5S, 18S and 28S ribosomal genes, and the smallest metacentric pair not labeled by our BACs (Figs. 4 and 7) [22, 24, 61]. In contrast, *Notothenia* species would mostly share fusions, characteristic of their reduced chromosomal number [25]. However, some fusions in *N. rossii* and in *N. coriiceps* could have appeared independently, as hinted by the different positions of the ribosomal genes either in a long arm, or in a short arm of sub-metacentric, probably non orthologous pairs [54, 79].

Positioning chromosomal fusions relative to speciation events is not easy for the *Trematomus* group given the multiple acquisition of rearrangement events and difficulties in establishing a reliable phylogeny for this radiation [15, 80]. Bursts of retrotransposon mobilization (including the *DIRS1* elements) possibly associated with environmental stress of the glaciation and deglaciation cycles on the Antarctic continental shelf have been proposed as a cause of genomic plasticity, facilitating chromosomal diversification [59, 60]. During mobilization of retrotransposons, DNA breaks and recombination may have occurred in different genomic locations, and thus involved different SUs and fusions in distinct populations. The process of speciation can last millions of years before two populations become completely isolated reproductively. Either ancestral or recent karyotypic polymorphisms are possible when taking into account the history and ecological context of the *Trematomus* adaptive radiation. Both alternatives would involve multiple independent fusions, and reinforce species isolation. Those fusions would then be randomly fixed in each of those diverging populations. In contrast, in the *Notothenia* group, fusions would mostly have been acquired before the *Notothenia*/*Paranotothenia* divergence (10–5 *My*, [14, 17] (Fig. 6). Indeed, the timing of diversification differs between the *Notothenia* and *Trematomus* groups. In contrast to the rapid diversification of genus *Trematomus* [15, 16, 80], the Nototheniinae is not considered as a radiation [7, 14, 19, 20, 70]. Fusions in the Nototheniinae might have had much more time to arise and become fixed in different populations before the next species divergence.

Even though *Trematomus* and *Notothenia* show two distinct and specific histories of fusion acquisition, the system may not be completely binary: in both groups, some fusions might have occurred early and some later. Further, large-scale studies would be needed to establish the timing of retrotransposon bursts and to identify and characterize rearrangement events in the different nototheniid groups to better understand these evolutionary histories and their link to speciation.

## Conclusions

We identified for the first time a large number of specific genomic regions involved in specific fusion event. We showed that large acrocentric, metacentric or sub-metacentric mitotic chromosomal pairs are indeed mostly products of chromosome fusions, which are the most frequent type of rearrangement event in the Nototheniidae [23, 57, 61]. We also demonstrated that the chromosomal segments (SUs) are strongly conserved across all notothenioids examined. Fusions involving multiple SUs that we examined are independent within the genus *Trematomus*, whereas they are shared among *Notothenia* species, illustrating two contrasting evolutionary histories of karyotype diversification within the family. These fusions may have accompanied the establishment of reproductive barriers between populations, or might have merely reinforced barriers initiated by other mechanisms. In either case, chromosomal fusions would be important actors in nototheniid speciation.

The increasing availability of nototheniid genome sequences and improved genome assemblies will enable the use of in silico genomic approaches to supplement cytogenomic tools for a comparative and integrated perspective, although experiments such as the ones described in this study are required to link genomics to physical chromosomes. Genome sequencing and assembly will improve both the resolution of chromosomal syntenies and the possibility to detect various types of chromosomal micro-rearrangements.

Divergence among nototheniid species is quite recent, so the chromosomal syntenies are highly conserved at the family scale. Even though the Southern Ocean is currently a highly stable environment, predicted perturbations including warming of about 0.1 °C per decade [81–84] are likely to have dramatic consequences on species evolution and development, with possible impacts on genome architecture and plasticity, and could deeply accelerate genomic changes and restructuring.

## Methods

### Fish specimens

Specimens of nineteen notothenioid species (*Bovichtus diacanthus*, *Chionodraco hamatus*, *Cygnodraco mawsoni*, *Dissostichus mawsoni*, *Eleginops maclovinus*, *Indonotothenia cyanobrancha, Gymnodraco acuticeps*, *Lepidonotothen larseni*, *Notothenia angustata*, *Notothenia coriiceps*, *Notothenia rossii*, *Patagonotothen ramsayi*, *Trematomus bernacchii*, *Trematomus borchgrevinki*, *Trematomus eulepidotus*, *Trematomus hansoni*, *Trematomus newnesi*, *Trematomus nicolai*, *Trematomus pennellii*) were collected during the French, Italian and American demersal fish survey programs. The chromosomal preparations and tissue samples for DNA extraction used are listed in Additional file 2.

### Chromosomal preparations

Mitotic chromosome preparations were obtained from primary cell cultures of pronephric kidney or spleen strictly following the specific protocol for Antarctic fish described in Rey et al. [85]. Fixed cell suspensions were preserved as aliquots of 15 ml at − 20 °C. Prior to use, suspensions were thawed and centrifuged at 1500 rpm (Centrifuge 5424R, Eppendorf, Hamburg, Germany) for 10 min at 4 °C. After removing supernatants, cell pellets were resuspended in 0.8–1 ml of fresh fixative (3:1 mix of cold ethanol/glacial acetic acid), and were spread onto Superfrost slides (pre-cleaned with absolute ethanol containing 1% of 1 N HCl) for BAC-FISH and chromosomal painting. For microdissection, cell suspensions were spread onto 40 × 60 mm coverslips and stained with GIEMSA (4%) for 12 min. Slides were stored at − 20 °C until further use. Additional file 2 summarizes the chromosomal preparations used in this study.

### Probes preparation

#### BAC probe selection

BAC clones used in this study were isolated from the *Notothenia coriiceps* VMRC-19 BAC library (average insert size 138 kb) [66–68]. Forty BACs were randomly selected from two 384-well plates (number 25 and 58). Four BACs (named 28D21, 25G6, 85H3, and 44 M14) were partially sequenced to determine their gene contents. The remaining 36 clones were not sequenced. The latter were named from their plate coordinates (with a • to differentiate the BACs extracted in plate number 58).

#### BAC amplification

BAC clones (138 kb average insert size) were amplified to provide genomic DNA for the BAC-FISH probes. For each BAC, 5 μl of its glycerol stock were inoculated into 5 ml of Lysogeny Broth (LB) culture medium containing chloramphenicol (12.5 μg/ml), and the mini-culture was incubated for 5 h at 37 °C with agitation (200 rpm, Incubating Orbital Shaker 12,620–946, VWR, Radnor, PA, USA). The culture was then transferred to 95 ml of enriched LB culture medium, and the midi-cultures were incubated for 18 h at 37 °C with agitation (200 rpm, Incubating Orbital Shaker 12,620–946, VWR). Once a culture attained an OD600 ≥ 0.6, BACs were purified using the Plasmid Midi prep kit (Qiagen, Hilden, Germany). BAC DNA was precipitated with ethanol and re-suspended in 30 μl of twice-distilled sterile water. BAC concentration was quantified using a Qubit fluorometer (Thermo Fisher Scientific, Waltham, MA, USA).

#### Chromosomal microdissection and preparation of the painting probe

A chromosome-specific probe of the largest sub-metacentric pair of *T. pennellii* was prepared by microdissection [86]. This chromosome pair is the only one that is easily distinguishable during dissection due to its large size (15 vs 5 μm on average). Glass microneedles were made using a PC-100 pipette puller (Narishige, Tokyo, Japan). Chromosome capture was performed using a Zeiss inverted microscope (Zeiss, Oberkochen, Germany) and a Narishige model number micromanipulator (Narishige model PC-10, Tokyo, Japan). Metaphases of interest were covered with 10 μl of sterile distilled water. The target chromosome pair was impaled by the needle tip and transferred to a 100 μl PCR tube containing 10 μl DNase-free water. After 30 chromosomal copies of the pair were collected, random DNA fragments were amplified by PCR using, 1) the Whole Genome Amplification WGA4 kit (Sigma-Aldrich, Saint-Louis, Mo, USA), followed by 2) the Whole Genome Reamplification WGA3 kit (Sigma-Aldrich) according to the manufacturer’s instructions. Amplified DNA was quantified by fluorometry using the Qubit dsDNA HS (high-sensitivity) array kit (Thermo Fisher Scientific). The WGA4 kit yielded ~ 4 μg of product from an input of 100 pg of chromosomal DNA, whereas the WGA3 kit gave ~ 7 μg of DNA from an input of 10 ng of WGA4 product.

#### Probe labeling

BAC clones (1 μg DNA) were labeled with fluorochromes (fluorescein-ULS, rhodamine-ULS or Dyomics415-ULS-dGTP) using PlatiniumBrightTM Nucleic Acid Labeling Kits (ULS 495-Green, ULS 550 Red/Orange, ULS 415 Blue; Leica Biosystems, Wetzlar, Germany) following the instructions provided by the supplier. Labeling was performed under low illumination to limit fading of the fluorochromes. Labeled BACs were precipitated using ethanol. BAC DNAs were collected by centrifugation (45 min at 13,500 rpm); and the DNA pellets were resuspended in hybridization buffer (50% formamide/2X SSC/10% dextran sulfate) for double or triple BAC-FISH (final probe concentrations = 16 ng/μl).

Chromosome-painting probes (1 μg DNA) were labeled with biotin-11-dUTP by random priming using the Biotin-High-Prime Kit (Roche Diagnostics, Meylan, France) according to the manufacturer’s instructions. Labeled painting probes were prepared in hybridization buffer to give a final concentration of 16 ng/μl as described for BAC clones.

#### Competitor and carrier DNAs

To prevent nonspecific hybridization of highly and moderately repetitive sequences present in the BAC and painting probes to chromosomes, we prepared genomic DNA from *N. coriiceps* and *T. hansoni* as specific competitors following Bonillo et al. [87]. As carrier, we used DNA from the red deer, *Cervus elaphus*. DNA was extracted from muscle tissue following Winnepenninckx et al. [88]. DNA was then fragmented by thermal shock. Following centrifugations, DNA pellets were resuspended in hybridization buffer (50% formamide/2X SSC/10% dextran sulfate) to final concentrations of 8 μg/μl (*N. coriiceps*, *T. hansoni*) or 10 μg/μl (*C. elaphus*).

#### Fish

BAC-FISH and chromosomal painting were performed on chromosome preparations from 19 notothenioid species (Additional file 2) according to Bonillo et al. [87]. Due to limited numbers of chromosomal spreads for some species, we tested the hybridization efficiency of two BACs (C4 and F15) to all species. Results demonstrated high cross-hybridization efficiency (Fig. 3).

Briefly, labeled BAC or painting probes were denatured by heating at 85 °C for 5 min in the presence of specific competitor and carrier DNA. Denatured BACs or painting probes were then incubated for 2 h at 37 °C with competitor DNAs before applying them to freshly thawed and denatured (72 °C, 10–60 s, 70% formamide/2X SSC) chromosome preparations. Slides were incubated for 48 h at 37 °C in a humid chamber, washed in appropriate buffers, and dehydrated by a succession of ethanol washes [87]. To ensure BAC and painting signal specificity, FISH was performed on chromosome preparations under high stringency washing conditions: 0.4X SSC, 0.3% Tween 20 at 60 °C for 2 min, followed by 2X SSC, 0.1% Tween 20 for 1 min. For both BAC and painting probes, DNA was counterstained with DAPI/antifade.

For chromosomal painting, slides were covered with 40 μl of FITC-avidin (Roche Diagnostics) under a 24 × 40 mm glass coverslip; incubated 5 min at 37 °C in a humid chamber, and washed three times in 4X SSC, 1% Tween 20 at room temperature for 2 min.

#### Image acquisition

FISH images were recorded using a Zeiss Axioplan microscope equipped with a cooled CCD camera (Coolsnap Photometrics, Tucson, AZ 85706, USA) and an XCite LED fluorescence light source. Karyotypes were processed using CytoVision 3.93.2/Genus FISH-imaging software for animal chromosomes (Leica Microsystems). Ten to 40 metaphase spreads per species were examined for each hybridized probe.

## Supplementary information

**Additional file 1.** Complete list of the 40 BACs studied. The BACs are grouped according to the signal category: “specific”, “repetitive” or “weak”. SU locations, chromosomal positions and species examined for “specific” BACs are indicated.

**Additional file 2.** Species sampling for chromosomal preparations and for tissue (muscle) for DNA extraction. This file contains a table describing all the specimens’ material used in this study.

## Data Availability

All data generated or analyzed during this study are included in this published article (and its supplementary information files). The *N. coriiceps* BAC library VMRC-19 [66–68] is available from J. H. Postlethwait and A. Amores on reasonable request.

## Abbreviations

AFGPs: Antifreeze glycoproteins
BAC: Bacterial artificial chromosome
DAPI: 4′,6-diamidino-2-phenylindole
FISH: Fluorescence in situ hybridization
FITC: Fluorescein isothiocyanate
ICHs: Interspecific chromosomal homologies
My: Million years
NORs: Nucleolus organizer regions
SU: Structural unit
TGD: Teleost genome duplication

## Footnotes

1 Throughout this publication we use the notothenioid nomenclature of Duhamel et al. [6], which designates the “Antarctic clade” as the family Nototheniidae, including 11 subfamilies (Fig. 7). This clade is synonymous with the AFGP-bearing notothenioids of Matschiner et al. [16] and the Cryonothenioidea of Near et al. [17].
