# Supplementary material for: Multiple independent chromosomal fusions accompanied the radiation of the Antarctic teleost genus Trematomus (Notothenioidei:Nototheniidae)
Source: BMC Evol Biol. 2020 Mar 20;20:39. doi: 10.1186/s12862-020-1600-3 (PMC7082932; doi:10.1186/s12862-020-1600-3)
Supplement: Supplementary file 1 — Additional file 1. Complete list of the 40 BACs studied. The BACs are grouped according to the signal category: “specific”, “repetitive” or “weak”. SU locations, chromosomal positions and species examined for “specific” BACs are indicated. [file 12862_2020_1600_MOESM1_ESM.pdf]

A)

**Specific signal**	**SU location - chromosomal position**	**Species examined**
25G6	SU10 (red) interstitial	Nco, Tne, Teu, Tni, Tpe, Tha, Tbe, Tbo
28D21	SU10 (red) interstitial	Nco, Nro, Nan, Tne, Teu, Tni, Tpe, Tha, Tbe, Tbo
44M14	SU11 (purple) interstitial or pericentromeric	Nco, Nro, Nan, Tne, Teu, Tpe, Tha, Tbe
85H3	SU9 (blue) telomeric	Nco, Tne, Teu, Tni, Tpe, Tha, Tbe, Tbo
C4	SU1 (blue) pericentromeric	Nco, Tne, Teu, Tni, Tpe, Tha, Tbe, Tbo, Icy, Lla, Pra, Cha, Cma, Dma, Gac, Dma
D2	SU7 (grey) interstitial	Nco
E21	SU13 (green) telomeric	Nco, Tne, Teu, Tni, Tpe, Tha, Tbe, Tbo, Icy
F3	SU9 (blue) interstitial	Tne, Teu, Tpe, Tha, Tbo
F5	SU13 (green) pericentromeric	Nco, Tne, Teu, Tni, Tpe, Tha, Tbe, Tbo, Icy
F7	SU2 (green) interstitial	Tne, Teu, Tpe, Tha, Tbo
F15	SU1 (blue) interstitial	Nco, Tne, Teu, Tni, Tpe, Tha, Tbe, Tbo, Icy, Lla, Pra, Cha, Cma, Dma, Gac, Dma
G6	SU10 (red) interstitial	Nco, Tne, Teu, Tni, Tpe, Tha, Tbe, Tbo
H12	SU1 (blue) pericentromeric	Nco
J8	SU3 (orange) pericentromeric	Nco, Tpe, Tha
K19	SU7 (grey) telomeric	Nco
M11	SU9 (blue) centromeric	Nco, Tne, Teu, Tni, Tpe, Tha, Tbe, Tbo
P3	SU6 (yellow) yellow	Nco, Tne, Teu, Tpe, Tha, Tbo
•F4	SU8 (brown) interstitial	Nco, Tpe, Tha
•F6	SU15 (pink) telomeric	Nco, Tpe
•F8	SU10 (red) pericentromeric	Nco, Tpe, Tha
•F10	SU6 (beige) interstitial	Nco
•F22	SU9 (blue) interstitial	Tpe, Tha

B)

Signal	Species examined
**Repetitive**	
B9	Nco
F11	
L17	
O24	
•F14	
•L2	
•O2	
**Weak**	
A10	Nco
C9	
D23	
E7	
F9	
I1	
N21	
•F12	
•F20	
•G2	
•J2	

Additional file 1: **Exhaustive list of BACs studied.** A) "Specific" refers to the twenty-two BACs (55% of total), which gave clearly discrete double spots at a single location on one chromosomal pair. For each of the "specific" BACs, SU locations (arbitrary number and corresponding color used in Fig 2 and 4), chromosomal positions and species examined are specified. B) "Repetitive" indicates a second BAC hybridization pattern observed for 7 BACs (17.5% of total); multiple signals on several chromosomal pairs, probably due to high proportion of repetitive sequences. "Weak" refers to the third signal category obtained with 11 BACs (27.5% of the total); single or multiple weak spots in nuclei that were rarely visible on metaphasic chromosomes (see Fig 1 for more details). These BACs were tested on *N. coriiceps* as part of the screening process, but not selected for the cross-hybridizations to *Trematomus* species.
