# Supplementary material for: Multiple independent chromosomal fusions accompanied the radiation of the Antarctic teleost genus Trematomus (Notothenioidei:Nototheniidae)
Source: BMC Evol Biol. 2020 Mar 20;20:39. doi: 10.1186/s12862-020-1600-3 (PMC7082932; doi:10.1186/s12862-020-1600-3)
Supplement: Supplementary file 2 — Additional file 2. Species sampling for chromosomal preparations and for tissue (muscle) for DNA extraction. This file contains a table describing all the specimens’ material used in this study. [file 12862_2020_1600_MOESM2_ESM.pdf]

sub-family or family	*Genus. Species*	Sampling type	Field reference	Locality
Trematominae	*T. pennellii*	chromosomes	CE4320[5]	Adelie Bank, Adelie Land margin
	T. newnesi	chromosomes	R6TA495[3] (M)	Adelie Bank, Adelie Land margin
	T. hansoni	chromosomes	CE4004[5] (F)	Adelie Bank, Adelie Land margin
		tissue	TA646TRHA2[3]	
	T. eulepidotus	chromosomes	M2S1	Ross Sea, Mario-Zuccheli station
	T. bernacchii	chromosomes	REVO2009-10 214-2738[5]	Adelie Bank, Adelie Land margin
	T. borchgrevinki	chromosomes	CE3588[5] (F)	Adelie Bank, Adelie Land margin
	T. nicolai	chromosomes	CE5684REVO856[5] (F)	Adelie Bank, Adelie Land margin
	I. cyanobrancha	chromosomes	PK9343[4]	Kerguelen-Heard Plateau
	L. larseni	chromosomes	LL12[2]	Shag rocks, South Atlantic
	P. ramsayi	chromosomes	PR1[2]	South Georgia
Nototheniinae	*N. coriiceps*	chromosomes	REVO2009-10lot316[5], TA15[3], TA25[3], TA335[3], TA731[3], NC5R2[2]	Adelie Bank, Adelie Land margin, Bouvet island
		tissue	REVO-080-270[5], REVO-080-272[5]	
	N. angustata	chromosomes	Nang1	Southern New-Zeland
	N. rossii	chromosomes	NR1	South Georgia
Dissostichinae	*D. mawsoni*	chromosomes	DM1	Ross Sea, Mario-Zucchelli station
Channichthyinae	*C. hamatus*	chromosomes	R60-II[1]	Ross Sea, Mario-Zucchelli station

Cygnodraconinae	*C. mawsoni*	chromosomes	TA373[3]	Adelie Bank, Adelie Land margin
Gymnodraconinae	*G. acuticeps*	chromosomes	TA736[3]	Adelie Bank, Adelie Land margin
Eleginopsidae	*E. maclovidus*	chromosomes	EM2[2]	Falkland Islands, Southern Ocean
Bovichtidae	*B. diacanthus*	chromosomes	BD4[2]	Tristan da Cunha Island, Southern Atlantic ocean

Additional file 2: **Species sampling for chromosomal preparations and for tissue (muscle) for DNA extraction.** Chromosomal preparations and tissue samples were collected during the French, Italian, American, and International Antarctic campaigns: **TNB**[1] (1990/1991, Zuchelli Station, Ross Sea), **ICEFISH**[2] (2004, Atlantic sector of the Southern Ocean), **ICOTA**[3] (1996-2008, Adelie Land), **POKER**[4] (2010 and 2013 Kerguelen-Heard shelf), **REVOLTA**[5] (2010-2014, Adélie Land). F stands for female and M for male when known.
